# Mitochondrial Protein PINK1 Positively Regulates RLR Signaling

**DOI:** 10.3389/fimmu.2019.01069

**Published:** 2019-05-14

**Authors:** Jun Zhou, Rui Yang, Zhaoru Zhang, Qianru Liu, Yuanyuan Zhang, Qingqing Wang, Hongbin Yuan

**Affiliations:** ^1^Department of Cell Biology, School of Basic Medical Sciences, Zhejiang University, Hangzhou, China; ^2^The Key Laboratory of Reproductive Genetics, Ministry of Education, Zhejiang University, Hangzhou, China; ^3^Department of Anesthesiology, Changzheng Hospital, Second Military Medical University, Shanghai, China; ^4^The Children's Hospital, Zhejiang University School of Medicine, Hangzhou, China; ^5^Institute of Immunology, Zhejiang University School of Medicine, Hangzhou, China

**Keywords:** PINK, RLR, TRAF3, ubiquitination, YAP1, antiviral immune response, macrophages, IRF3

## Abstract

The serine/threonine kinase phosphatase and tensin homolog (PTEN)-induced putative kinase 1(PINK1) controls mitochondrial quality and plays a vital role in the pathogenesis of early-onset Parkinson's disease. However, whether PINK1 has functions in innate antiviral immunity is largely unknown. Here, we report that viral infection down regulates PINK1 expression in macrophages. PINK1 knockdown results in decreased cytokine production and attenuated IRF3 and NF-κB activation upon viral infection. PINK1 promotes the retinoic-acid-inducible gene I (RIG-I)-like receptors (RLR)-triggered immune responses in a kinase domain-dependent manner. Furthermore, PINK1 associates with TRAF3 via the kinase domain and inhibits Parkin-mediated TRAF3 K48-linked proteasomal degradation. In addition, PINK1 interacts with Yes-associated protein 1 (YAP1) upon viral infection and impairs YAP1/IRF3 complex formation. Collectively, our results demonstrate that PINK1 positively regulates RIG-I triggered innate immune responses by inhibiting TRAF3 degradation and relieving YAP-mediated inhibition of the cellular antiviral response.

## Introduction

As the host's first line of defense, innate immunity detects, and fights against pathogen invasion. The recognition of viral infection by the innate immune system depends on germline-encoded pattern-recognition receptors, including TLRs (Toll-like receptors), RLRs (retinoic-acid-inducible gene I (RIG-I)-like receptors), Nod-like receptors, and DNA sensors (1, 2). TLRs (TLR3, TLR7/8, and TLR9) recognize virus-derived RNA and DNA in the endosome. The RLR members RIG-I and MDA5 (melanoma differentiation-associated gene 5) sense viral RNAs in the cytoplasm, whereas cGAS (cyclic GMP-AMP synthase) is responsible for the recognition of viral DNA in the cytoplasm (3, 4).

RIG-I, the most important RLR family member, recognizes viral RNA such as vesicular stomatitis virus (VSV), respiratory syncytial virus (RSV), and Sendai virus (SeV) and induces IFN-β production (5). After recognition of invading viruses, RIG-I recruits adaptors, including MAVS, TRAF3, and results in TRAF3 ubiquitination, which provides docking sites for the formation of the TBK1/IKKε complex. The activated complex subsequently phosphorylates the transcription factors IRF3/IRF7 and induces the nuclear translocation of IRF3/IRF7 dimers to trigger type I IFNs and proinflammatory cytokine production (6). Aberrant RLR signaling is associated with autoimmune and/or inflammatory diseases such as systemic lupus erythematosus (3), chronic obstructive pulmonary disease (COPD) (7); hence, moderate activation of RLRs signaling is critical for efficient viral clearance without harmful immunopathology.

PINK1 [phosphatase and tensin homolog (PTEN)-induced putative kinase 1] is a serine/threonine kinase that is responsible for the pathogenesis of early-onset Parkinson's disease (PD) (8). PINK1 can act upstream of Parkin to remove damaged mitochondria via mitophagy (9). Aside from its role in neurodegeneration and mitophagy, PINK1 has multiple distinct functions in regulating cell metabolism, cancer development and inflammation (10). PINK1 can promote hepatic insulin resistance (IR) via JNK and ERK signaling in palmitate (PA)-treated HepG2 cells (11). PINK1 suppresses MDV (mitochondrial-derived vesicles) formation and MitAP (mitochondrial antigen presentation) provoked by inflammation (12). PINK1 has also been implicated in liver inflammation due to hepatitis B (HBV) or C virus (HCV) infection (13–15). In addition, a review discussed the neurological sequelae of infection by viruses known to induce parkinsonism, including influenza virus, Coxsackie, Japanese encephalitis B, and HIV viruses (16). Recent epidemiologic studies have revealed that patients with HCV infection might be at an increased risk of PD (17, 18). These data suggest that virus infection might be involved in the pathogenesis of PD. However, the role of PINK1 in antiviral immunity has not been reported.

Here, we report that viral infection downregulates PINK1 expression in macrophages. PINK1 enhances RLR-triggered type I interferon and proinflammatory cytokine production in a kinase domain dependent manner. PINK1 associates with TRAF3, inhibiting TRAF3 proteasomal degradation after conjugation to K48-linked ubiquitin by Parkin. Furthermore, PINK1 inhibits the interaction between Yes-associated protein 1 (YAP1) and IRF3, leading to increased IRF3 activation. Our data demonstrate that PINK1 functions as a positive regulator of the antiviral immune response by regulating TRAF3 degradation and YAP1/IRF3 complex formation.

## Materials and Methods

### Cells and Virus Infection

RAW264.7 macrophages and HEK293T cells were obtained from the American Type Culture Collection (ATCC, Rockville, MD, USA) and maintained in DMEM supplemented with 10% fetal bovine serum (FBS). For stable cell lines, RAW264.7 macrophages were transfected with jetPRIME reagent (Polyplus) according to manufacturer's instructions, and then were selected with puromycin (8 μg/ml) and pooled for further experiments. For mouse peritoneal macrophages, C57BL/6 mice (6–8 weeks old) were intraperitoneally injected with thioglycollate (Sigma). Three days later, abdominal cells were collected and cultured in RPMI-1640 medium with 10% FBS, then adhered peritoneal macrophages were subjected for successive experiments. Mice were housed in pathogen-free conditions. All animal experiments were approved by the Animal Review Committee of Zhejiang University School of Medicine and were in compliance with institutional guidelines.

Primary macrophages were infected with RSV (kindly provided by Dr. Qingqing Wang, Zhejiang University School of Medicine, MOI = 10), VSV (MOI = 1), and herpes simplex virus (HSV) (MOI = 10) (kindly provided by Dr. Xiaojian Wang, Zhejiang University School of Medicine). HEK293T cells were infected with VSV (MOI = 0.1). MOIs were selected as described previously (19). The cells or supernatants were harvested for immunoblotting assays or ELISA. All experiments using viruses were conducted in a biosecurity level 2 laboratory approved by School of Basic Medical Sciences, Zhejiang University.

### Plasmids, Transfection, and RNA Interference

Plasmids expressing PINK1 (RC206970), Parkin (RC221147), and TRAF3 (RC201106) were purchased from Origene Technologies (Rockville, MD, USA). The vector of these plasmids is pCMV6 Entry with C-terminal Myc/DDK (Flag) tags. Mutants PINK1ΔKD and PINK1 L347P were amplified by PCR from full-length PINK1 cDNA using a MutanBest kit (TaKaRa). Plasmids expressing HA-IRF3, GFP-TRAF3, HA-Ub and Flag-YAP1 were provided by Dr. Huazhang An (Second Military Medical University, Shanghai). The plasmids were transfected into RAW264.7 and HEK293T cells using jetPEI (Polyplus, Illkirch, France). PINK1- specific siRNA was transfected into primary macrophages using Lipofectamine RNAiMAX (Thermo Fisher Scientific, Waltham, MA, USA) according to the standard protocol. PINK1 siRNA (5′- CCA GGC GGU AAU UGA CUA TT-3′, 5′- UAG UCA AUU ACC GCA CUG GTT-3′) was from GenePharma (Shanghai, China).

### Generation of PINK1 Knockout Cells

CRISP/Cas9 guide RNA targeting sequence for mouse Pink1 was designed using the MIT online tool (http://crisp.mit.edu). The CRISPR plasmid pEP-330x (kindly provided by Dr. Xiaojian Wang, Zhejiang University School of Medicine) contains expression cassettes of Cas9 and puromycin resistant gene. gRNA targeting the exons of PINK1 was GAGGTCACTGCTCCAGCGAG and inserted into the pEP-330x vector, and then co-transfected into RAW264.7 cells using jetPEI for 48 h. Then puromycin (8 μg/ml) (Sigma-Aldrich) was used for selection in RAW264.7 cells. Individual clones were isolated by limiting dilution cloning, and knockout of PINK1 was confirmed via western blotting.

### Antibodies

Antibodies against phosphorylated and total IRF3, IKKε, TBK1, NF-κB p65, ERK, JNK, and p38 were purchased from Cell Signaling Technology (Danvers, MA, USA). Anti-TRAF3, anti-β-actin, and HRP-conjugated secondary antibodies were from Santa Cruz. Anti-Myc, anti-Flag, anti-HA, and anti-GFP were from Origene. Anti-PINK1, anti-Parkin, and anti-YAP1 were from Abcam Biotechnology (Cambridge, UK). Anti-K48-ubiquitin and anti-K63-ubiquitin were from Millipore (Kenilworth, USA).

### RNA Isolation and Real-Time Quantitative PCR (Q-PCR)

Total RNA was isolated from cells with TRIzol reagent (Takara) following the manufacturer's directions. cDNA was generated from total RNA using reverse transcriptase (Takara). SYBR RT-PCR kits (Takara) were used for quantitative real-time PCR analysis as described (20). The primers used for mRNA analysis are listed in Table 1. Gapdh and β-actin were used as housekeeping genes for human and mouse samples, respectively. Quantitative normalization of target mRNA expression was performed for each sample using housekeeping gene expression as an internal control.

**Table 1:** Primers for Real-time quantitative PCR.

Human pink1 forward primer	5^′^- CAAGAGAGGTCCCAAGCAAC−3^′^
Human pink1 reverse primer	5^′^- GGCAGCACATCAGGGTAGTC−3^′^
Human gapdh forward primer	5^′^-ATTCCACCCATGGCAAATTC-3^′^
Human gapdh reverse primer	5^′^-GGATCTCGCTCCTGCAAGATG-3^′^
Mouse pink1 forward primer	5^′^- GAGCAGACTCCCAGTTCTCG−3^′^
Mouse pink1 reverse primer	5^′^-GTCCCACTCCACAAGGATGT−3^′^
Mouse actin forward primer	5^′^- AGTGTGACGTTGACATCCGT-3^′^
Mouse actin reverse primer	5^′^-GCAGCTCAGTAACAGTCCGC-3^′^
Mouse il-6 forward primer	5^′^-TAGTCCTTCCTACCCCAATTTCC-3^′^
Mouse il-6 reverse primer	5^′^-TTGGTCCTTAGCCACTCCTTC-3^′^
Mouse IFN-β forward primer	5^′^- ATGAGTGGTGGTTGCAGGC−3^′^
Mouse IFN-β reverse primer	5^′^- TGACCTTTCAAATGCAGTAGATTCA−3^′^
VSV-G forward primer	5^′^- ACGGCGTACTTCCAGATGG-3^′^
VSV-G reverse primer	5^′^- CTCGGTTCAAGATCCAGGT-3^′^


### ELISA

Cell supernatants were collected and mouse Il-6 (eBioscience, Thermo Fisher Scientific) and IFN-β (Biolegend, San Diego, CA) levels were determined according to manufacturer's instructions. The ELISA plates were read on a microplate reader. The results were calculated as the difference between the absorbance at 570 nm and the absorbance at 450 nm. Quantification was performed according to the standard curve as described in the manufacturer's instructions.

### Immunoblotting and Immunoprecipitation

Cells were lysed using cell lysis buffer containing protease inhibitor “cocktail” (Cell Signaling Technology). The protein concentrations in the extracts were measured using the BCA assay (Thermo Fisher Scientific). For immunoblotting, equal amounts of extracts were separated by SDS-PAGE, transferred onto polyvinylidene fluoride membranes (Millipore), and then probed with the indicated antibodies. For immunoprecipitation, the supernatants were incubated overnight with specific antibodies at 4°C overnight, then incubated with protein A/G Sepharose (sc-2003, Santa Cruz) for another 2 h. The beads were washed four times with cold PBS containing 0.05% Tween-20, and immunoprecipitates were eluted with loading buffer. β-actin levels in total cell lysates were measured to show equal protein loading.

### Immunofluorescence Staining

HEK293T cells grown on coverslips were infected or uninfected with VSV at 4 h. To label mitochondria, 100 nM MitoTracker™ Red CMXRos (Invitrogen) was added to the medium for 25 min at 37°C. Cells were washed with PBS and fixed with 2% paraformaldehyde, permeabilized with 0.1% Triton X-100, blocked with 5% BSA, and stained with mouse anti-PINK1 and rabbit anti-TRAF3, anti-YAP1 antibody, followed stained with Alexa Fluor 488 anti-mouse and Alexa Fluor 594 anti-rabbit secondary antibody, to detect co-localization of PINK1 with TRAF3, YAP1, or Mitochondria. The co-localization was detected with Zeiss LSM 880 with AiryScan.

### Human Peripheral Blood Samples

A total of 38 peripheral blood samples of bronchiolitis with RSV infection were collected from Children's Hospital, Zhejiang University School of Medicine, China. An additional 21 control blood samples were obtained from healthy children. RSV infection was confirmed by RSV antigen tests of nasopharyngeal aspirates. Other antigens (influenza virus, parainfluenza virus, metapneumovirus, and adenovirus antigens) or microbiological tests, including blood cultures, protein-purified derivative test, and serology for Chlamydia pneumonia, Mycoplasma pneumonia, and Legionella pneumophila, were performed to exclude other common respiratory tract infection and tuberculosis. Detailed patient information is shown in Tables 2, 3. The ethics committee of Children's Hospital, Zhejiang University School of Medicine approved the study. Written informed consent was obtained from at least one guardian for each patient before enrollment.

**Table 2:** Basic information of human peripheral blood samples about healthy control and pediatric patients with RSV infection from Children's Hospital.

	**Normal** **control *n* = 21**	**RSV patients** *n***= 38**
**AGE(MONTHS)**		
≤3	10	26
3–6	6	7
≥6	5	5
**GENDER**		
Male	14	28
Female	7	10

*Twenty-one control blood samples were obtained from healthy children. A total of 38 peripheral blood samples of bronchiolitis with RSV infection were collected from Children's Hospital*.

**Table 3:** The detailed information of RSV-infected patients.

**Number**	**Gender**	**Age**	**Grade of infection**	**RSV antigen test**	**Clinical symptom**		
					**Cough**	**Short of breath**	**Fever**
1	Male	1M24D	Mild	+	+	+	–
2	Female	2M10D	Mild	+	+	+	–
3	Female	6M13D	Mild	+	+	+	–
4	Female	2M26D	Mild	+	+	+	–
5	Female	3M19D	Mild	+	+	+	–
6	Male	6M8D	Mild	+	+	+	+
7	Female	3M5D	Mild	+	+	-	–
8	Male	1M11D	Mild	+	+	+	–
9	Male	4M18D	Mild	+	+	+	–
10	Male	3M3D	Mild	+	+	+	–
11	Male	2M3D	Mild	+	+	+	–
12	Male	8M17D	Mild	+	+	+	–
13	Male	9M9D	Mild	+	+	+	–
14	Male	2M11D	Mild	+	+	+	–
15	Male	9M8D	Mild	+	+	+	–
16	Male	2M8D	Mild	+	+	+	–
17	Male	2M21D	Mild	+	+	+	–
18	Male	1M4D	Mild	+	+	+	–
19	Female	1M8D	Mild	+	+	-	–
20	Male	4M2D	Mild	+	+	+	–
21	Female	1M28D	Moderate	+	+	+	–
22	Male	2M20D	Moderate	+	+	+	–
23	Male	2M2D	Moderate	+	+	+	+
24	Male	2M5D	Moderate	+	+	+	–
25	Male	2M7D	Moderate	+	+	+	–
26	Male	2M29D	Moderate	+	+	+	–
27	Male	1M4D	Moderate	+	+	+	–
28	Male	4M4D	Moderate	+	+	+	–
29	Female	4M21D	Moderate	+	+	+	–
30	Male	2M29D	Moderate	+	+	+	–
31	Male	1M10D	Moderate	+	+	+	–
32	Male	1M20D	Moderate	+	+	+	–
33	Female	1M23D	Moderate	+	+	+	–
34	Male	1M14D	Moderate	+	+	+	–
35	Male	1M14D	Moderate	+	+	+	–
36	Male	1M4D	Severe	+	+	+	–
37	Male	2M24D	Severe	+	+	+	+
38	Female	1M21D	Severe	+	+	+	+

*Additional information for all patients*:

*In the age group, “Y” is year, “M” is month, “D” is day*.

*The tests for other viral antigens are negative, including influenza virus, parainfluenza virus, metapneumovirus, and adenovirus*.

*The tests for other respiratory tract infection, including tuberculosis, Chlamydia pneumoniae, Mycoplasma pneumonia, and Legionella pneumophila are negative*.

Peripheral blood mononuclear cells (PBMCs) were isolated by Ficoll density gradient centrifugation (Sigma) following the manufacturer's instructions, stored at −80°C as cell pellets until RNA isolation was performed. The data were analyzed anonymously.

### Statistical Analysis

Statistical significance between groups was determined using two-tailed Student's *t*-test and two-way ANOVA. *P*-values of <0.05 were considered statistically significant.

## Results

### RNA and DNA Viral Infection Down Regulates PINK1 Expression in Macrophages

**Figure 1:**
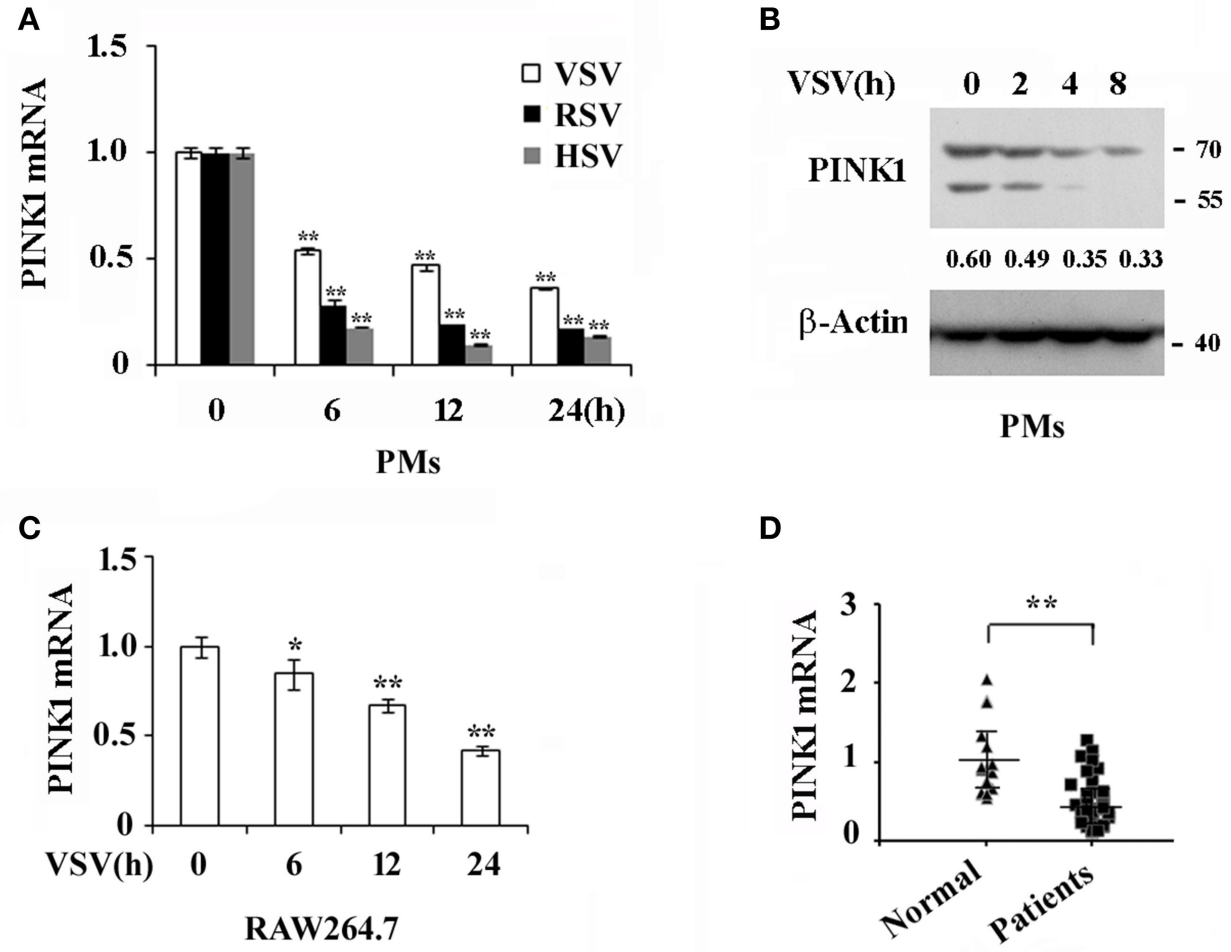
Virus infection down regulates PINK1 expression in macrophages. **(A)** Q-PCR analysis of PINK1 mRNA expression in mouse peritoneal macrophages (PMs) (3 × 10^5^) infected with VSV (MOI = 1), RSV (MOI = 10), or HSV (MOI = 10), respectively, for indicated hours. **(B)** Immunoblot analysis of PINK1 protein expression in mouse peritoneal macrophages (3 × 10^6^) infected with VSV for indicated hours. Numbers below lanes (top) indicate densitometry of the presented protein relative to β-Actin expression in that same lane (below). **(C)** Q-PCR analysis of PINK1 mRNA expression in RAW264.7 cells (1 × 10^5^) infected with VSV for indicated hours. **(D)** Q-PCR analysis of PINK1 mRNA expression in peripheral blood mononuclear cells (PBMCs) of 38 pediatric patients with RSV infection and 21 healthy children. Data are mean ± SD. Similar results were obtained in three independent experiments.*p < 0.05, **p < 0.01, compared with control.

To investigate whether PINK1 is involved in host antiviral innate immune response, we detected PINK1 expression in primary mouse peritoneal macrophages (PMs) infected with RNA viruses, including VSV and RSV, and DNA virus, HSV. As shown in Figure 1A, PINK1 mRNA expression was down-regulated after VSV, RSV, or HSV infection in PMs. Consistent with the mRNA result, Western blotting showed that PINK1 protein expression was attenuated upon VSV infection (Figure 1B). Moreover, PINK1 expression was decreased in RAW264.7 macrophage cell line (Figure 1C). We further recruited a cohort of peripheral blood samples from 38 pediatric patients with RSV infection and 21 healthy control children from Children's Hospital of Zhejiang University School of Medicine. Downregulated PINK1 mRNA expression was also observed in peripheral blood mononuclear cells (PBMCs) from patients compared with that in the healthy group (Figure 1D). Taken together, these data indicated that PINK1 expression might be correlated with the host antiviral immune response.

### PINK1 Knockdown Inhibits Virus-Triggered Cytokines Production in Macrophages

**Figure 2:**
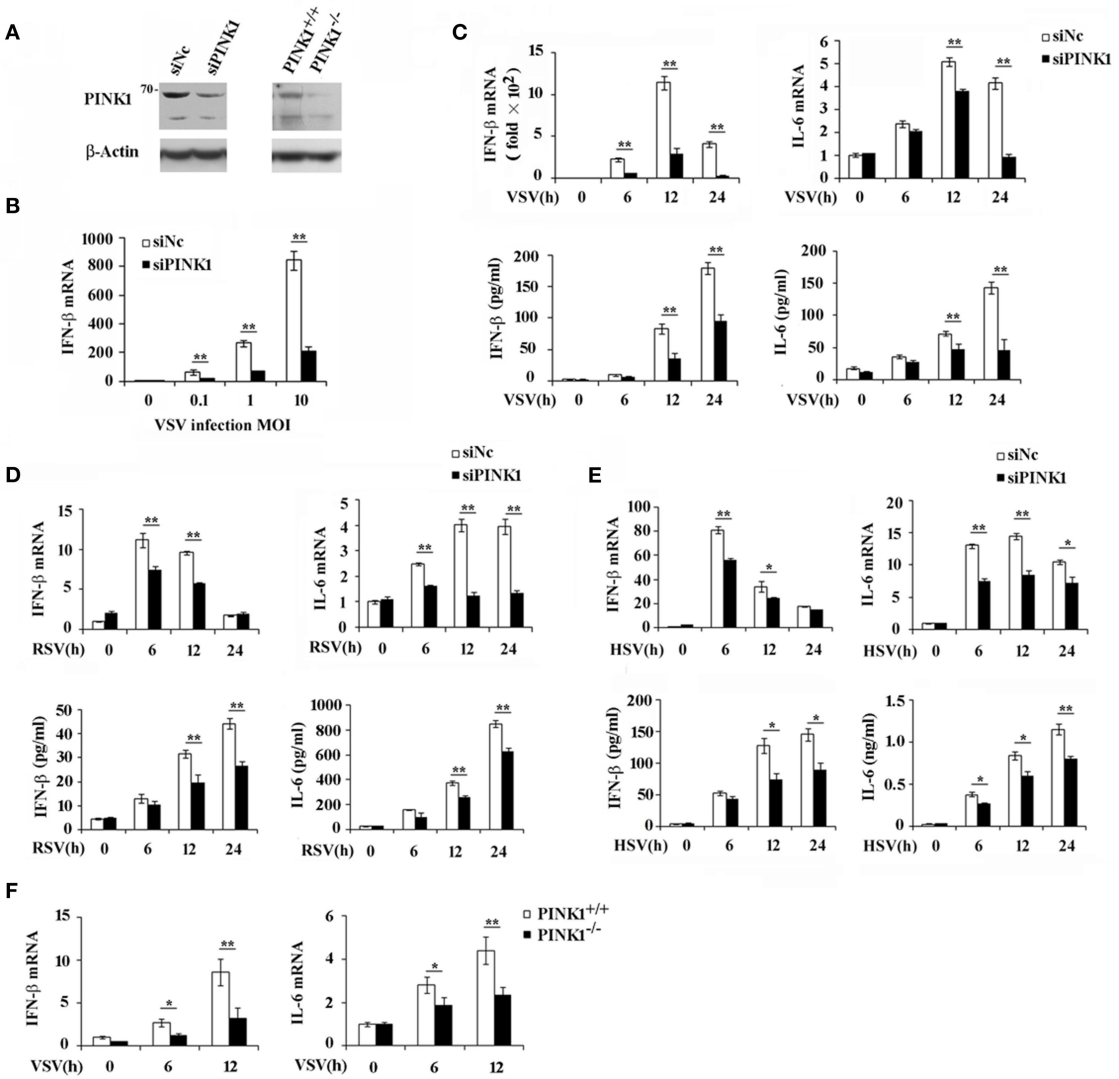
PINK1 knockdown or knockout suppresses virus-induced type I interferon and proinflammatory cytokine production. Mouse peritoneal macrophages (PMs) were transfected with 30 nM scrambled negative control siRNA (siNC) or PINK1 siRNA (siPINK1) for 48 h. PINK1 knockout RAW264.7 macrophages (PINK1^−/−^) were generated using CRISPR/Cas9 gene-editing system. **(A)** Immunoblot analysis of PINK1 expression level in PMs with PINK1 knockdown, or RAW264.7 cells with PINK1 knockout. **(B)** qPCR analysis of IFN-β mRNA expression in PMs infected for indicated MOIs with VSV for 6 h. **(C–E)** qPCR and ELISA analysis of IFN-β and IL-6 levels of in PMs infected with VSV, RSV, or HSV, respectively, for indicated hours. **(F)** qPCR analysis of IFN-β and IL-6 levels in wild type (PINK1^+/+^) and PINK1 knockout cells (PINK1^−/−^) RAW264.7 cells infected with VSV for indicated hours. Data are mean ± SD and are representative of three independent results. *p < 0.05, **p < 0.01, compared with control.

To investigate the role and functional significance of PINK1 in the host antiviral innate immune response, we silenced PINK1 expression with small interfering RNA in mouse peritoneal macrophages, followed by infecting with different viruses. Western blotting confirmed that PINK1 expression was significantly downregulated in macrophages transfected with PINK1-specific siRNA (Figure 2A). QPCR and ELISA analysis revealed that IFN-β expression was significantly decreased after VSV infection. Proinflammatory cytokine IL-6 expression was also downregulated in PINK1-knockdown macrophages infected with VSV (Figure 2B). Infection with different VSV (MOI) doses in macrophages induced similar decreases in IFN-β expression (Figure 2C). In addition, downregulation of IFN-β and IL-6 expression in PINK1-silenced macrophages was validated by QPCR and ELISA analysis in macrophages infected with another RNA virus, RSV, and a DNA virus, HSV (Figures 2D,E). Furthermore, infection with VSV in PINK1 knockout macrophages showed similar statistically significant decreases in IFN-β and IL-6 expression (Figure 2F). These data demonstrated that PINK1 knockdown suppressed virus-induced type I interferon and proinflammatory cytokine production. We therefore focused on the regulatory role of PINK1 in RNA virus-induced innate immune response.

### PINK1 Promotes RLR-Triggered IRF3 and NF-κB Activation

**Figure 3:**
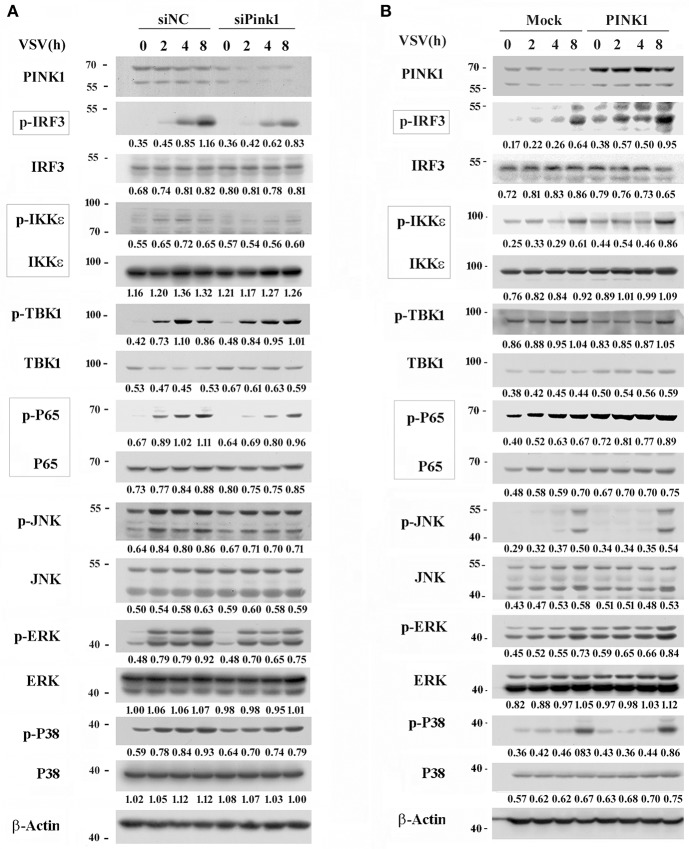
PINK1 promotes RLR-triggered IRF3 and NF-κB activation in macrophages. Mouse peritoneal macrophages transfected with 30 nM scrambled negative control siRNA (siNC) or PINK1 siRNA **(A)**, or RAW264.7 cells transfected with plasmids encoding Myc-PINK1 **(B)**, were infected with VSV for indicated hours. Phosphorylated or total proteins in lysates were detected by western blot. Numbers below lanes (top) indicate densitometry of the presented protein relative to β-Actin expression in that same lane (below). Data are representative of three independent experiments.

Upon RNA virus infection, transcription factors such as IRF3, NF-κB, and ATF2-c-Jun are activated and recruited to initiate type I interferon and proinflammatory cytokine transcription (21, 22). To elucidate the underlying mechanism by which PINK1 mediates RNA virus-induced cytokines production, we observed the effect of PINK1 knockdown and overexpression on IRF3 and NF-κB activation in macrophages. PINK1-specific siRNA significantly inhibited VSV-induced phosphorylation of IRF3, NF-κB subunit p65, and upstream IKKε in peritoneal macrophages. TBK1 phosphorylation was not affected by PINK1 knockdown. However, downregulation of p65 and IKKε might partly result from decreased p65 and IKKε total protein expression (Figure 3A). Consistent with these results, IRF3, p65, and IKKε phosphorylation was enhanced in PINK1-overexpressing RAW264.7 cells compared with control cells (Figure 3B). The mitogen-activated protein kinases JNK and p38 mediate activation of the ATF2-c-Jun heteodimer in the virus-induced cytokines response (21). Pink1 knockdown slightly inhibited the VSV-induced MAPK activation. However, MAPK phosphorylation except ERK was not significantly affected by PINK1 overexpression in macrophages (Figures 3A,B). These data demonstrated that PINK1 might mediate RLR-triggered immune response by regulating molecules upstream of IRF3 and NF-κB.

### PINK1 Associates With TRAF3 and IRF3 After RLR Activation

**Figure 4:**
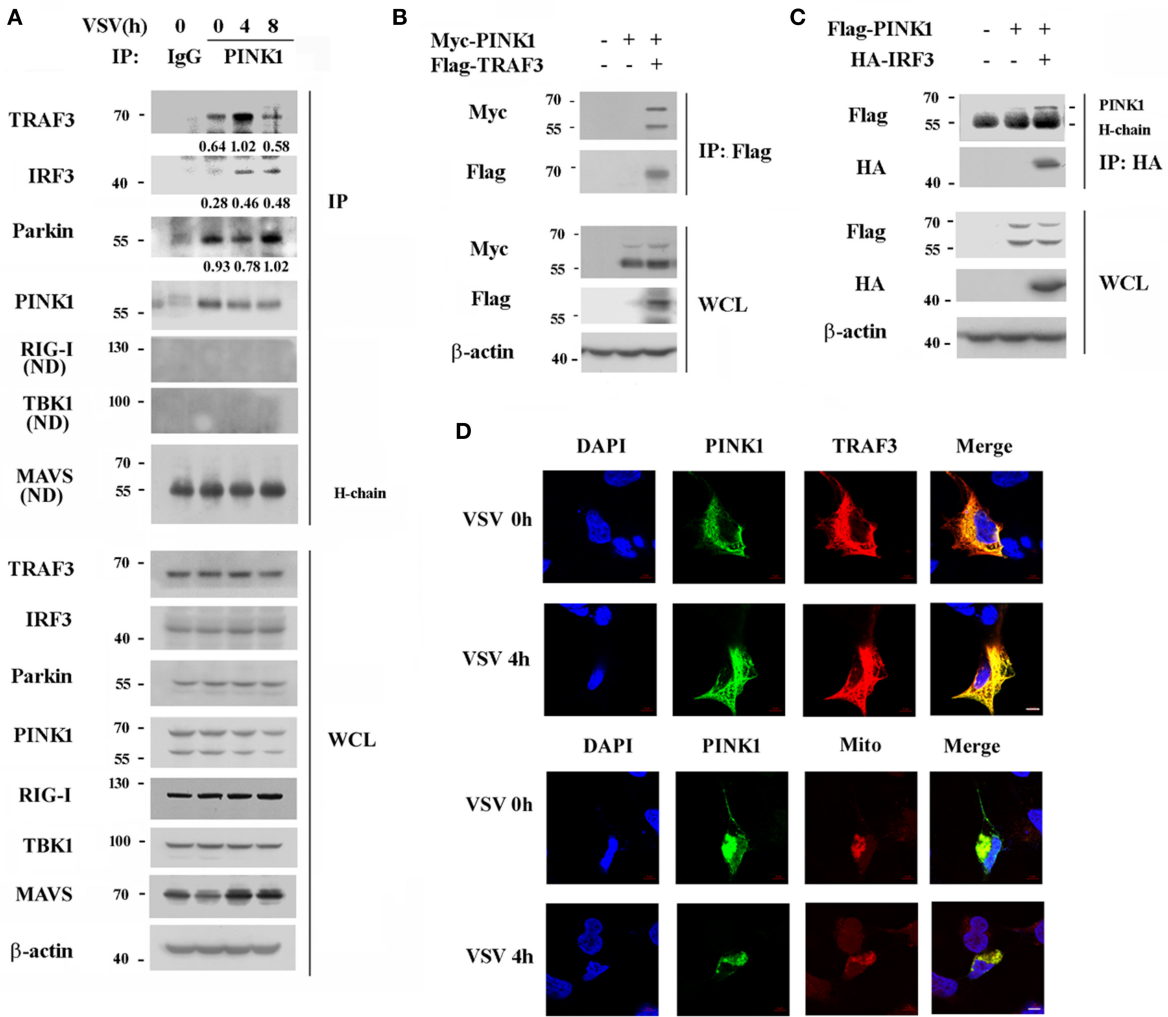
PINK1 interacts with TRAF3 and IRF3 upon VSV infection. **(A)** Mouse peritoneal macrophages were infected with VSV for indicated hours. Immunoblot analysis of endogenous TRAF3, IRF3, Parkin, RIG-I, TBK1, and MAVS immunoprecipitated with antibody to PINK1. IgG was as control. Numbers between two blots indicate densitometry of TRAF3, IRF3, or Parkin relative to that of PINK1 in immunoprecipitates. **(B,C)** HEK293T cells were transfected with PINK1 expressing plasmid together with Flag-TRAF3 or HA-IRF3 plasmid. Cells were lysated 24 h after transfection for immunoblot analysis of indicated proteins immunoprecipitated with antibody to Flag **(B)** or HA **(C)** tag. **(D)** Confocal microscopy of HEK293T co-transfected with Myc-PINK1 and Flag-TRAF3 plasmids followed by VSV infection for 4 h. MitoTracker (Mito) was used to probe the mitochondrion (red). DAPI served as a marker of nuclei (blue). Scale bar, 5 μm. Data are representative of three independent experiments.

To further investigate the underlying mechanisms by which PINK1 positively regulates RIG-I triggered signaling, we investigated potential PINK1 target proteins in the RIG-I signaling pathway in mouse peritoneal macrophages. The primary upstream signal adaptors of RIG-I signaling, such as RIG-I, MAVS, TRAF3, TBK1, IRF3, were detected in immune complexes precipitated with an anti-PINK1 antibody. PINK1 physically interacted with endogenous TRAF3 in resting primary mouse peritoneal macrophages, and this interaction was enhanced upon VSV infection, whereas the interaction between PINK1 and IRF3 was only detected after VSV infection. In addition, the association of PINK1 with Parkin, an E3 ubiquitin-ligase, was detected in both resting and stimulating macrophages. However, PINK1 did not detectably associate with RIG-I, MAVS, or TBK1 (Figure 4A). We further detected the interaction between PINK1 and TRAF3 or IRF3 in HEK293 cells. Consistent with the result in macrophages, exogenously expressed Myc-PINK1 efficiently interacted with Flag-TRAF3 or HA-IRF3 (Figures 4B,C). The co-localization of PINK1 and TRAF3 was observed in the cytosol in both VSV uninfected or infected HEK293T cells. Furthermore, subcellular co-localization analysis showed that PINK1 colocalized with the motichondrial marker (MitoTracker). Additionally, viral infection had no significant effect on the co-localization of PINK1 and mitochondria (Figure 4D). Collectively, these results indicated that PINK1 regulated RLR-triggered IRF3 and NF-κB activation possibly by targeting TRAF3 and IRF3, and PINK1 colocalized with TARF3 in the cytosol mitochondria.

### PINK1 Promotes RLR-Triggered Immune Response via Kinase Domain Dependent Manner

**Figure 5:**
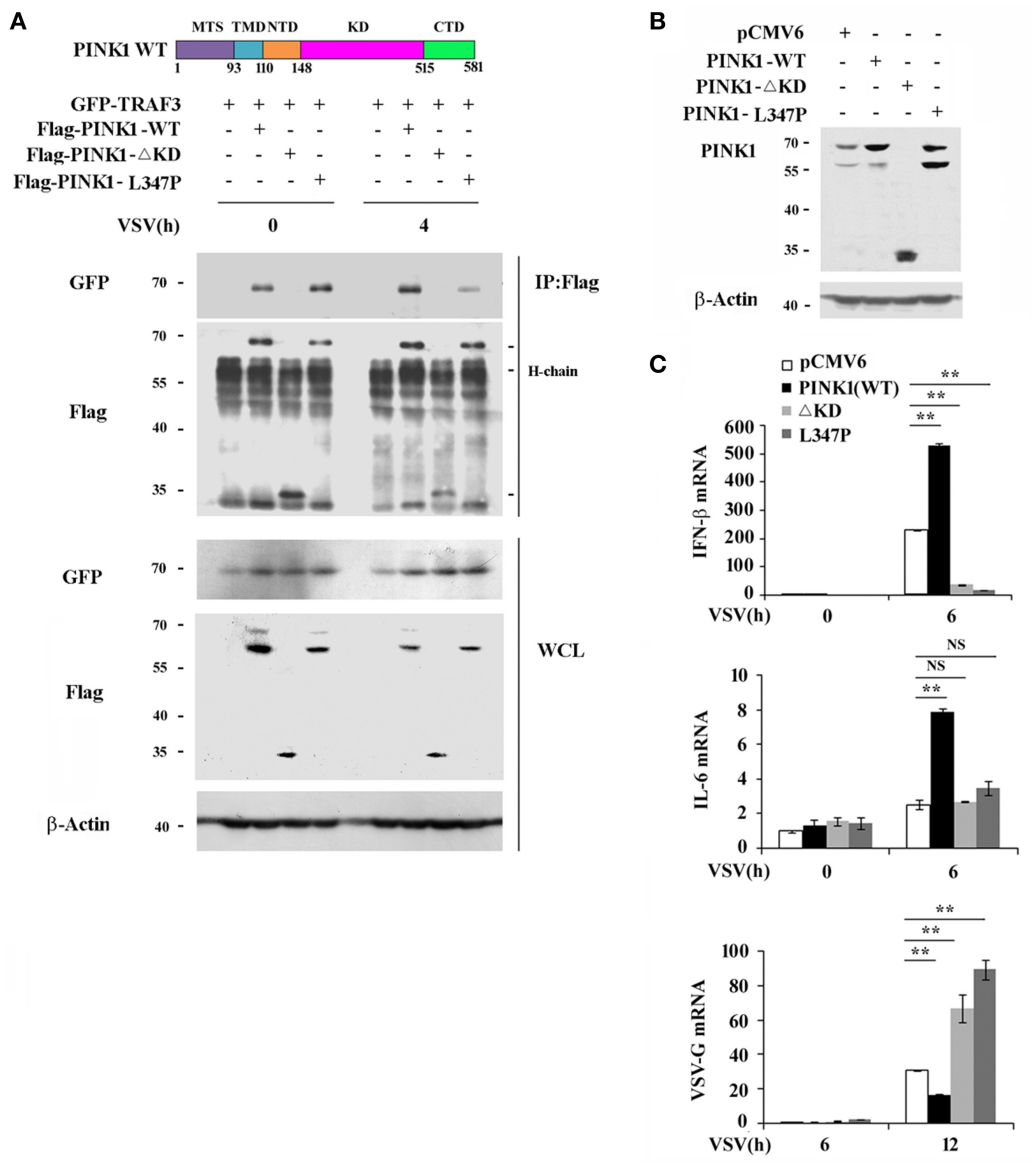
PINK1 promotes RLR-triggered immune response via kinase domain dependent manner. **(A)** Immunoblot analysis of HEK293T cells that cotransfected with GFP-TRAF3 plus Flag-PINK1, Flag-PINK1 mutants vectors followed by VSV infection for 4 h, then immunoprecipitated with antibody to Flag tag. **(B)** Immunoblot analysis of RAW264.7 cells stably overexpressed with empty vector or vector encoding PINK1 and its mutants. **(C)** qPCR analysis of IFN-β, IL-6, and VSV-G mRNA in stably transfected RAW264.7 cells expression of PINK1 variants, infected with VSV for indicated times. Data are representative of three independent experiments. **p < 0.01.

PINK1 contains an N-terminal mitochondrial targeting sequence (MTS), a transmembrane domain (TMD), and a central highly conserved serine-threonine kinase domain (KD). To determine whether PINK1 interacts with TRAF3 and promotes RLR-triggered immune response via its kinase domain, we constructed two mutations: PINK1ΔKD, which lacks kinase domain and PINK1 L347P, which is reported to exhibit low protein stability and reduced kinase activity in cells (23, 24). Binding analysis revealed that both PINK1 wild type (WT) and PINK1 L347P interacted with TRAF3 in HEK293T cells in a VSV-infection independent manner, whereas the kinase domain-deleted PINK1ΔKD mutant did not (Figure 5A). PINK1(c-tagged) WT plasmid shows an intact band of full length protein at about 70 kDa and a major degradation product at about 60 kDa. L347P mutation mainly shows degradation band (23). PINK1 WT stable RAW264.7 cells mainly express 70 kDa band, whereas L347P mutation stable cells express much more 60 kDa degradation band (Figure 5B). Furthermore, the antiviral function dependent on the induced expression of type I interferon and proinflammatory cytokine by PINK1 was abolished in both PINK1-mutated cells (Figures 5B,C).Taken together, these data suggested that PINK1 promoted RLR-triggered immune response in a kinase domain-dependent manner.

### PINK1 Inhibits Parkin-Mediated K48-Linked TRAF3 Ubiquitination

**Figure 6:**
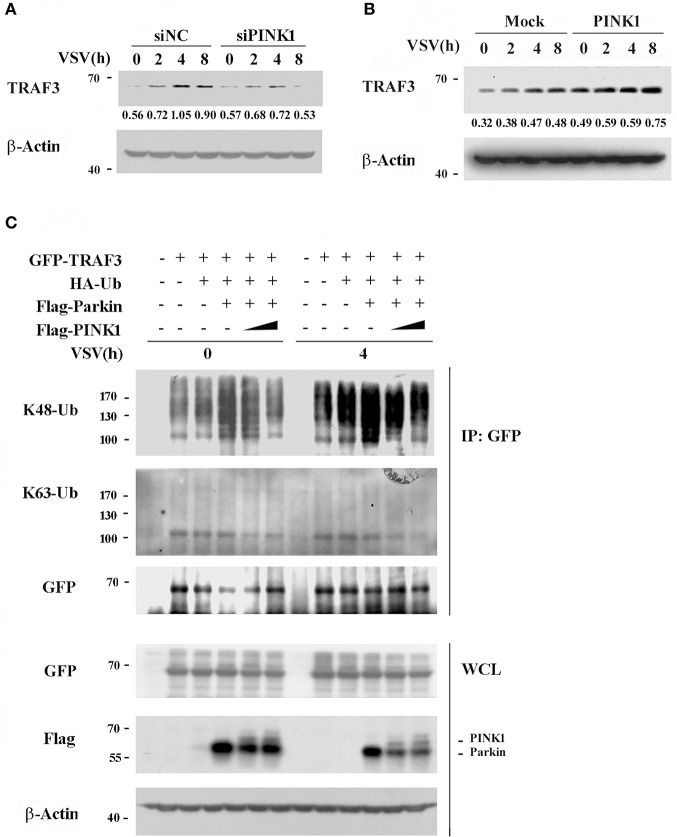
PINK1 inhibits TRAF3 degradation. Mouse peritoneal macrophages transfected with scrambled negative control siRNA (siNC) or PINK1 specific siRNA (30 nM) **(A)**, or RAW264.7 cells transfected with PINK1 plasmid **(B)**, were infected with VSV for indicated hours. TRAF3 proteins in lysates were detected by western blot. Numbers below lanes (top) indicate densitometry of the presented protein relative to β-Actin expression in that same lane (below). **(C)** HEK293T cells were transfected with GFP-TRAF3, HA-Ub, Flag-Parkin, and varying doses of Flag-PINK1 (0, 0.5, and 1 μg) and infected with VSV for 4 h. Cells were treated with MG132 (10 uM) and harvested for immunoblot analysis of K48-Ub and K63-Ub immunoprecipitated with antibody to GFP tag. Data are representative of three independent experiments.

Ubiquitination is a versatile posttranslational modification that plays important roles in antiviral immune response. TRAF3 is reported to be modified with a poly-ubiquitin chain to provide a scaffold for adaptor complex formation in RIG-I signaling (25, 26). PINK1 plays neuroprotective roles against dysfunctional mitochondria by recruiting Parkin, a cytosolic E3 ubiquitin ligase (27). Based on the interaction of PINK1 with TRAF3 and Parkin, we analyzed the effect of PINK1 on TRAF3 expression and ubiquitination. We detected the effect of PINK1 expression on TRAF3 protein expression in response to VSV infection. PINK1 knockdown resulted in lower TRAF3 expression in mouse peritoneal macrophages following VSV infection (Figure 6A). Consistent with the knockdown results, PINK1 overexpression yielded higher basal TRAF3 expression levels and increased TRAF3 protein expression in RAW264.7 macrophages after VSV infection (Figure 6B). Considering that PINK1 interacts with Parkin in a VSV infection-independent manner in macrophages (Figure 4A), we investigated whether PINK1 regulates TRAF3 expression via ubiquitination. Compared with transfection using empty vector, Parkin overexpression increased the K48-linked ubiquitination of TRAF3 in both resting and VSV-infected HEK293 cells. However, PINK1 overexpression caused substantial loss of K48 ubiquitination in a dose-dependent manner. Parkin did not affect K63-linked ubiquitination (Figure 6C). At the same time, the presence of PINK1 downregulated Parkin expression (Figure 6C). These results suggested that PINK1 regulated TRAF3 protein levels through K48-linked proteasomal degradation, likely due to reduced Parkin expression.

### PINK1 Inhibits the Interaction of YAP1 With IRF3

**Figure 7:**
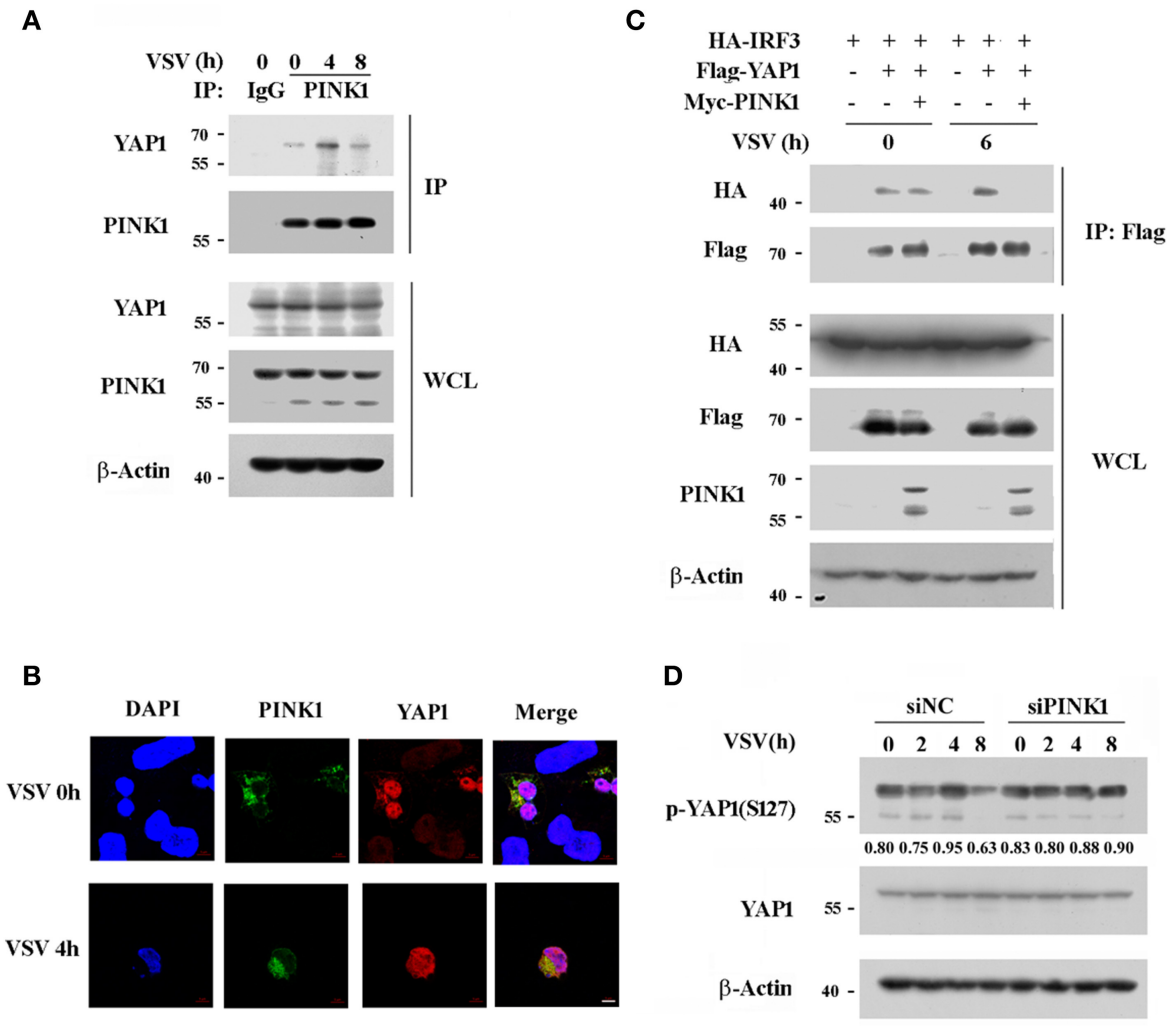
PINK1 associates with YAP1 and inhibits the interaction of YAP1 with IRF3. **(A)** Mouse peritoneal macrophages were infected with VSV for indicated hours. Immunoblot analysis of endogenous YAP1 immunoprecipitated with antibody to PINK1. IgG was as control. **(B)** Confocal microscopy of HEK293T cells that co-transfected with Myc-PINK1 and Flag-YAP1 plasmids followed by VSV infection for 4 h. Scale bar, 5 μm. **(C)** HEK293T cells were transfected for 24 h with plasmids encoding HA-IRF3, Flag-YAP1, and Myc-PINK1. Immunoblot analysis of indicated proteins immunoprecipitated with antibody to Flag tag. **(D)** Mouse peritoneal macrophages transfected with scrambled negative control siRNA (siNC) or PINK1 specific siRNA (30 nM) were infected with VSV for indicated hours. Phosphorylated or total proteins of YAP1 in lysates were detected by western blot. Similar results were obtained in three independent experiments.

PINK1 is reported to be a positive regulator of cell cycle progression (28), while YAP1 is a transcriptional activator of the Hippo signaling pathway, which promotes cell growth and inhibits apoptosis (29). Recently, YAP1 was identified as a negative regulator of innate immunity by interacting with IRF3 to impair dimmer formation and nuclear translocation after viral infection (30). This finding led us to investigate whether PINK1 regulates the RIG-I pathway via YAP1 upon RNA virus infection. PINK1 interacted with endogenous YAP1 in resting macrophage, and the association was significantly increased by VSV infection for 4 h (Figure 7A). Immunoflurescence analysis showed that PINK1 colocalized with YAP1 in both resting and VSV infected HEK293T cells (Figure 7B). Further study revealed that PINK1 did not interfere with the interaction between YAP1 and IRF3 in resting HEK293 cells. The exogenous YAP1/IRF3 complex was more easily detected in HEK293 cells 6 h after infection with VSV. However, the association was almost undetectable in cells overexpressing PINK1 (Figure 7C). In addition, PINK1 knockdown only affected YAP1 phosphorylation at Ser127 after VSV infection for 8 h (Figure 7D). These findings demonstrated that PINK1 positively regulated RIG-I signaling at least partly by impairing the interaction of YAP1 with IRF3 and liberating IRF3 from YAP1-mediated inhibition.

## Discussion

**Figure 8:**
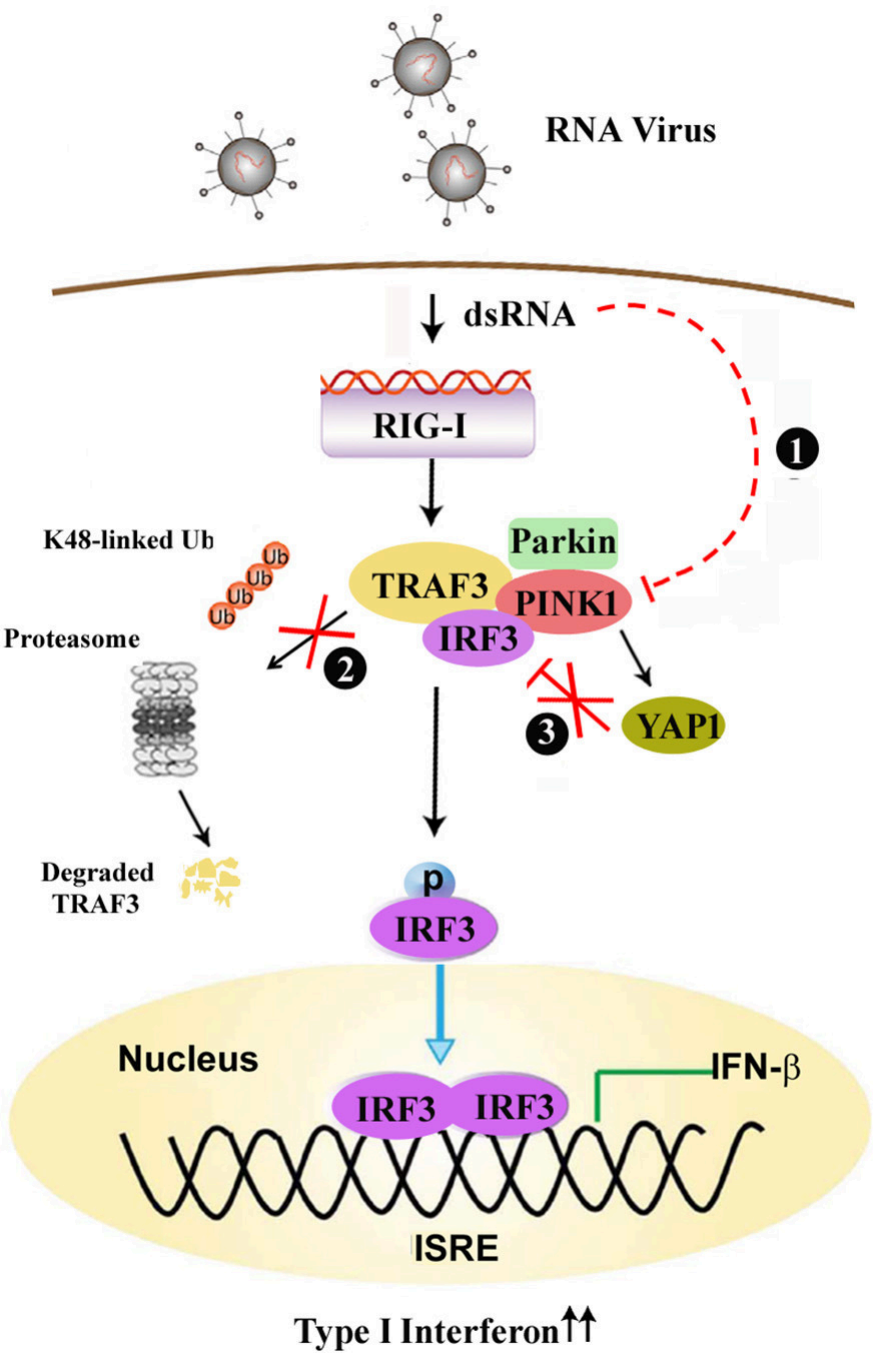
PINK1 positively regulates the RLR-triggered antiviral immune response by inhibiting TRAF3 degradation and relieving YAP-mediated inhibition of the cellular antiviral response. (1) Virus infection down-regulates PINK1 expression in macrophages. (2) PINK1 promotes RLR-triggered IRF3 activation via inhibiting Parkin-mediated K48-linked ubiquitination of TRAF3, (3) and PINK1 inhibits YAP1/IRF3 complex formation, which ultimately promotes RIG-I triggered antiviral immune response.

Mutations in PINK1 and Parkin are the two most common causes of early-onset, recessively inherited PD (8, 31). Immune dysregulation, including the upregulation of inflammatory gene expression, has long been considered a hallmark of PD. Numerous viruses can enter the nervous system and induce a variety of encephalopathies, including parkinsonism (16). However, the precise role of PINK1 in antiviral innate immune responses, as well as its crosstalk with the TLR, RLR signaling pathway is poorly understood. In the present study, we have shown that PINK1 positively regulates the RLR-triggered antiviral immune response by inhibiting TRAF3 degradation and YAP1/IRF3 complex formation (Figure 8). To our knowledge, this is the first report to link PINK1 and RLR signaling.

Viruses use different strategies to escape host antiviral immunity and support persistent viral infection and spread (32). Some IAV (Influenza A virus) strains control innate antiviral defense by binding to Riplet and inhibiting Lys63-linked RIG-I polyubiquitylation by NS1 protein (33). An epidemic strain of DENV (Dengue virus) isolated in Puerto Rico has been shown to sustain RIG-I signaling by binding to TRIM25 in a sequence-specific manner and inhibiting its deubiquitylation (34). Here we observed decreased PINK1 expression in PBMCs from pediatric patients infected with RSV compared with those from healthy children. PINK1 expression was also downregulated in primary macrophages infected with DNA and RNA viruses. These data suggest that PINK1 expression might be correlated with the host antiviral immune response.

TRAF3 plays a pivotal role in RLR-triggered IRF3 activation and subsequent type I interferon and proinflammatory cytokine production (35). PINK1 positively regulates the antiviral immune response through enhanced association with TRAF3 and IRF3 upon virus infection, which promotes IRF3 phosphorylation and results in increased IFN-β and IL-6 transcription. Furthermore, PINK1 associates with TRAF3 and promotes RIG-I triggered cytokine transcription in a kinase domain-dependent manner. However, macrophages overexpressing the ΔKD and L347P PINK1 variants displayed much lower IFN-β expression and subsequently much higher VSV-G mRNA compared with control cells. PINK1 mutants might reduce E3 ligase activity, leading to increased susceptibility to stress and accumulation of un/misfolded proteins in cells, eventually leading to cell death (36). Thus, the lower IFN-β expression in PINK1 mutant-overexpressing cells upon VSV infection might be associated with un/misfolded proteins such as Parkin recruitment and cell death. Further studies are therefore needed to examine the cell viability of macrophages overexpressing PINK1 mutants.

Ubiquitination of adaptor proteins is one of the most versatile posttranslational regulations and is widely involved in the precise modulation of antiviral response activity (26, 37). E3 ubiquitin ligases, such as TRIM23, TRIM29, were involved in posttranslational modification of NEMO or MAVS by ubiquitination to regulate antiviral immune response (38–40). TRAF3 maintains its activity in a suitable state by undergoing K48- and K63-linked ubiquitination after viral stimulation (41, 42). We detected an association between PINK1 and TRAF3 in resting macrophages, which was enhanced upon VSV infection. However, PINK1 does not belong to any known ubiquitin ligase families. In the mitophagy pathway, the ubiquitin-like domain (UBL) of Parkin is a crucial substrate of PINK1 that is phosphorylated at Ser65 (43, 44). Recently, Parkin was reported to be a negative regulator of antiviral signaling pathway by targeting TRAF3 for degradation (45). Xiong et al. reported that PINK1 expression not only regulates Parkin E3 ligase activity but also promotes the degradation of Parkin substrates, including Parkin itself (36). Our results showed that PINK1 promoted TRAF3 expression upon viral infection, likely due to PINK1-mediated Parkin degradation and repressed Parkin-mediated TRAF3 K48-linked ubiquitination, leading to decreased TRAF3 degradation. Our data suggest that PINK1 regulate TRAF3 degradation in response to viral stimulus, possibly by promoting Parkin degradation, to precisely modulate the immune response.

YAP functions as a transcriptional regulator of organ-size control and tissue homeostasis (46). Park2 (Parkin) was identified as a YAP target gene and was upregulated in SalvCKO TRAP-seq (47). Hippo deficient cardiomyocytes showed increased expression of stress response genes including the quality control gene of mitochondria, Park2 (Parkin) (48). Wang et al. reported that YAP can negatively regulate antiviral innate immunity by interacting with IRF3 and impairing IRF3 dimer formation and subsequent nuclear translocation (30). Considering that PINK1 binds IRF3 after viral infection in macrophages, we speculate that YAP1 might be involved in the PINK1-mediated antiviral innate immune response. In this study, we found that PINK1 disrupted the binding between YAP1 and IRF3 following VSV infection. PINK1 knockdown only affect the phosphorylation of YAP at Ser127 at 8 h upon VSV infection (Figure 3C). YAP phosphorylation at Ser403 is critical for IKKε-mediated YAP lysosomal degradation and dissociation of YAP from IRF3 (30). Whether PINK1 affect YAP1 phosphorylation at Ser403 and thus impair the interaction between YAP1 and IRF3 is worthy of further study. Our data suggest that PINK1 promotes VSV-triggered RLR signaling at least partly by inhibiting the association of YAP1 and IRF3.

In conclusion, our data offer insights into PINK1 and Parkin function in antiviral innate immune response. In addition to mitochondrial quality control, PINK1 positively regulates the RIG-I triggered innate immune response by inhibiting TRAF3 degradation and relieving YAP-mediated inhibition of the cellular antiviral response.

## Ethics Statement

Human subjects: This study was carried out in accordance with the recommendations of institutional guidelines, the ethics committee of Children's Hospital, Zhejiang University School of Medicine with written informed consent from guardians of all subjects. At least one guardian for each subject gave written informed consent in accordance with the Declaration of Helsinki. The protocol was approved by the ethics committee of Children's Hospital, Zhejiang University School of Medicine.

Animal subjects: This study was carried out in accordance with the recommendations of institutional guidelines, the Animal Review Committee of Zhejiang University School of Medicine. The protocol was approved by the Animal Review Committee of Zhejiang University School of Medicine.

## Author Contributions

JZ and HY designed and supervised the research. JZ, RY, and ZZ conducted the experiments. QL, YZ, and QW analyzed the data. JZ wrote the manuscript.

### Conflict of Interest Statement

The authors declare that the research was conducted in the absence of any commercial or financial relationships that could be construed as a potential conflict of interest.
